# No association between in utero exposure to emissions from a coalmine fire and post-natal lung function

**DOI:** 10.1186/s12890-023-02414-7

**Published:** 2023-04-14

**Authors:** Emily J. Hemstock, Rachel E. Foong, Graham L. Hall, Amanda J. Wheeler, Shyamali C. Dharmage, Marita Dalton, Grant J. Williamson, Caroline Gao, Michael J. Abramson, Fay H. Johnston, Graeme R. Zosky

**Affiliations:** 1 Menzies Institute for Medical Research, University of Tasmania, Hobart, TAS Australia; 2 Centre for Air Pollution, Energy and Health Research, NHMRC CRE, Glebe, NSW Australia; 3 Children’s Lung Health, Wal-Yan Respiratory Research Centre, Telethon Kids Institute, Nedlands, WA Australia; 4 School of Allied Health, Curtin University, Bentley, WA Australia; 5 Commonwealth Scientific and Industrial Research Organization, Aspendale, VIC, Australia; 6 Allergy and Lung Health Unit, School of Population and Global Health, University of Melbourne, Melbourne, VIC Australia; 7 School of Public Health & Preventive Medicine, Monash University, Melbourne, VIC Australia; 8 Orygen Centre for Youth Mental Health, University of Melbourne, Parkville, VIC Australia; 9 Tasmanian School of Medicine, University of Tasmania, Hobart, TAS Australia

**Keywords:** Particulate matter, Respiratory function, Early life, Long-term effects, In utero exposure

## Abstract

**Background and objective:**

Studies linking early life exposure to air pollution and subsequent impaired lung health have focused on chronic, low-level exposures in urban settings. We aimed to determine whether in utero exposure to an acute, high-intensity air pollution episode impaired lung function 7-years later.

**Method:**

We conducted a prospective cohort study of children who lived in the vicinity of a coalmine fire. Respiratory function was measured using the forced oscillation technique (FOT). Z-scores for resistance at 5 Hz (R_5_), reactance at 5 Hz (X_5_) and area under the reactance curve (AX) were calculated. Two sets of analyses were conducted to address two separate questions: (1) whether mine fire exposure (a binary indicator; conceived after the mine fire vs in utero exposed) was associated with the respiratory Z-scores; (2) whether there was any dose–response relationship between fire-related PM_2.5_ exposure and respiratory outcomes among those exposed.

**Results:**

Acceptable lung function measurements were obtained from 79 children; 25 unexposed and 54 exposed in utero. Median (interquartile range) for daily average and peak PM_2.5_ for the exposed children were 4.2 (2.6 – 14.2) and 88 (52—225) µg/m^3^ respectively. There were no detectable differences in Z-scores between unexposed and exposed children. There were no associations between respiratory Z-scores and in utero exposure to PM_2.5_ (daily average or peak).

**Conclusion:**

There was no detectable effect of in utero exposure to PM_2.5_ from a local coalmine fire on post-natal lung function 7-years later. However, statistical power was limited.

**Supplementary Information:**

The online version contains supplementary material available at 10.1186/s12890-023-02414-7.

## Summary at a glance

The coalmine fire in Hazelwood (Victoria, Australia) was an extreme air pollution episode that lasted for 6-weeks leading to community concern regarding the potential health effects. We found no detectable effect of in utero exposure to PM_2.5_ from the fire on post-natal lung function, 7-years later.

## Introduction

Air pollution accounts for ~ 4.2 million deaths annually [1] and has been consistently associated with respiratory morbidity in adult populations [1, 2]. Particulate matter (PM) is a major component of air pollution that contains a range of reactive compounds [3, 4]. PM with an aerodynamic diameter of < 2.5 µm (PM_2.5_) can penetrate to the peripheral regions of the lung [5] and is strongly associated with respiratory morbidity and mortality [1, 2].

Gestation is a window of vulnerability for a developing foetus and maternal exposure to toxicants during this time can have lifelong consequences for the offspring [6, 7]. Higher PM_2.5_ exposure during pregnancy is associated with small birth weight, intrauterine growth restriction, pre-term birth and reduced lung function [6], [7]. The respiratory system begins developing at ~ 4 weeks gestation and reaches a critical point of air space expansion and surfactant production between 24 weeks’ gestation and birth [6]. Exposure to environmental pollutants during this period may lead to deficits in lung development that manifest as impaired lung function post-natally and an increased risk of disease later in life [6–8].

A number of studies have investigated the relationship between prenatal PM_2.5_ exposure and lung function in childhood. These studies have found inconsistent associations between prenatal exposure to PM_2.5_ and reduced lung function in children under 10 years of age [9–11], and no associations at 10 to 15 years of age [12, 13]. In addition, most of these studies have been conducted in the context of chronic exposure to PM_2.5_ which has made it difficult to separate ongoing effects of post-natal exposure to PM, from in utero effects. This has limited our ability to predict the health consequences of prenatal exposure to extreme air pollution events.

Hazelwood was an open-cut coal mine located in the Latrobe Valley in Eastern Australia. In 2014, embers from a landscape fire caused the coal seam to ignite and burn continuously for 45 days [14]. In Australia, the NEPM (National Environmental Protection Measure; https://www.nepc.gov.au/nepms/ambient-air-quality) air quality standard for PM_2.5_ is 25 µg/m^3^ as a 24-h average. Out of the 45 days the fire burned, our modelling suggests the PM_2.5_ standard was exceeded on 23 days and reached a maximum PM_2.5_ of 731 µg/m^3^ in the town of Morwell [14, 15]. The Early-Life Follow-up (ELF) [16] stream of the Hazelwood Health Study (HHS) has previously shown that children exposed post-natally to emissions from this fire had mild impairments in peripheral lung mechanics, 3 years after the fire [15]. As the children who were in utero at the time of the fire were not old enough to reliably conduct lung function measurements at the first follow-up, the 7-year follow-up was our first opportunity to investigate the impact of in utero exposure from this fire on respiratory health.

The aim of this study was to investigate the association between exposure to PM_2.5_ emitted from the coalmine fire and lung function in a cohort of children, who were only exposed in utero.

## Methods

### Participants

**Fig. 1:**
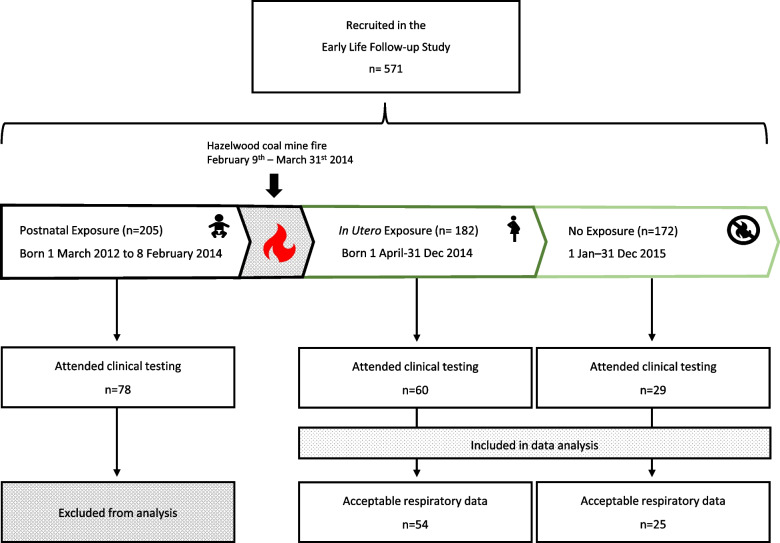
Flow chart of children participating in the study. Enrolment, exposure category, attendance and acceptable respiratory measurements of unexposed and in utero exposed children

The study sample was derived from a cohort of 571 children born between March 1^st^, 2012 and December 31^st^, 2015 in the Latrobe Valley who were in utero at the time of the fire, from February 9^th^, 2014 – March 31^st^, 2014. Unexposed children were conceived after the fire was completely extinguished, from January 1^st^, 2015 – December 31^st^, 2015. Children were recruited between February and September 2016, and the participating parent/carer completed a baseline survey with sociodemographic, health and family information. A detailed diary with the geographic location of each participant every 12 h during the fire was included, this was converted into a Statistical Area Level 1 location and used in the exposure assessment (see below). All 571 participants were invited to participate in a clinical follow-up study 7-years after the coalmine fire, between March and July, 2021. Only children with acceptable respiratory measurements and classified as unexposed or exposed (in utero) were included (Fig. 1).

All studies were approved by the Tasmanian Health and Medical Human Research Ethics Committee (reference H0014875). Additional approval was received from the Human Research Ethics Committees of Monash University, Monash Health and the University of Melbourne. All parents and caregivers of the participants provided signed informed consent.

### Exposure estimate

Air quality monitoring started 4 days after the fire and was conducted at multiple locations for varying time periods. Air quality was monitored by ambient air quality, PM monitoring and a roving PM monitoring system was used to assess the impact of smoke across the local region. These emissions were used in a prognostic meteorological, dispersion and chemical transport model with local wind data assimilation [14, 17, 18]. The model estimated PM_2.5_ emission rates at hourly time intervals based on the area of coal burned with a 1 × 1 km spatial resolution as described previously [14, 17]. Individual exposure estimates were then calculated for each 24-h period, based on the location diaries completed by the parents every 12-h. Modelled background ambient PM_2.5_ was subtracted from these estimates to provide daily average mean and maximum PM_2.5_ exposure estimates.

### Respiratory function

Lung function was evaluated using the forced oscillation technique (FOT) (TremoFlo C-100, Thorasys, Montreal, QC, Canada) according to American Thoracic Society/European Respiratory Society guidelines [19]. Standardized Z-scores were calculated for resistance (R_5_) and reactance (X_5_) at a frequency of 5 Hz and the area under the reactance curve (AX) [20]. The Z-scores account for differences in age, height, and gender [20].

### Statistical analysis

Two sets of analyses were conducted to address two separate questions: (1) whether mine fire exposure (a binary indicator; conceived after the mine fire vs in utero exposed) was associated with the respiratory Z-scores; (2) whether there were any dose–response relationships between fire-related PM_2.5_ exposure (mean daily PM_2.5_ and maximum daily PM_2.5_) and respiratory outcomes among those exposed (children conceived after the mine fire were excluded).

Univariate linear regression models were first used to evaluate the association between exposure to mine fire PM and respiratory Z-scores (R_5_, X_5_, AX). Mean average daily PM was assessed in increments of 10 µg/m^3^ and maximum average daily PM was assessed in increments of 100 µg/m^3^. Possible covariates were identified *a priori* based on our previous work and included; sex, BMI, breastfeeding duration, maternal education, smoking during pregnancy, alcohol consumption during pregnancy, overall stress, fire stress, maternal asthma, exposure to second-hand smoke, cold or flu or medication usage in the last 24 h [15]. However, due to the limited sample size, inclusion of all of these covariates resulted in overfitting. To deal with this, step-wise regression models (using R package olsrr version 0.5.3) were used to select covariates for each individual outcome variable to develop the final multivariate regression model. Multiple Imputation by Chained Equations (MICE using the mice R package version 3.14.0) with predicted mean matching was used to address missing data in outcome variables and covariates. The imputed datasets were used in stepwise variable selection. Covariates were selected if they were retained in 80% of stepwise models across imputed datasets and pooled with Rubin’s rule to calculate estimated regression coefficients in the linear regression models (Table S3, Supporting Information) [21].

R Studio Version 4.1.3 was used for the statistical analysis (Table S3, Supporting Information). Summary data are reported as means (SD) or median with ranges. Beta (β)-coefficients, 95% confidence intervals and *p* values are reported for all regression analyses.

## Results

### Participant characteristics

Of the 89 children from the unexposed or exposed groups who attended the clinic, 79 had acceptable respiratory measurements according to ARS/ERS criteria (Fig. 1). As expected, the exposed children were significantly older (*p* < 0.001), and taller (*p* < 0.001), than the unexposed children (Table 1). Interestingly, the unexposed children were also born at a younger gestational age (*p* = 0.005) and a smaller birthweight (*p* = 0.01) (Table 1).

**Table 1:** Participant characteristics and covariates of children that had acceptable respiratory measurements

	**Unexposed *n* = 25**	**In Utero exposure *n* = 54**	**Comparison of groups**
	mean ± sd (range)	mean ± sd (range)	*P*-value
Age (years)	6.0 ± 0.4 (5.4 – 7.1)	6.8 ± 0.3 (6.4 – 7.3)	** < 0.001**
Height (cm)	117.3 ± 5.6 (107.1 – 129.5)	123.0 ± 5.2 (110.0 – 133.5)	** < 0.001**
Weight (kg)	22.9 ± 4.1 (18.9 – 35.9)	25.4 ± 5.5 (19.2 – 44.7)	**0.041**
**Covariate characteristics**			
IRSD decile	3.6 ± 3.1 (1.0 – 9.0)	3.3 ± 2.6 (1.0 – 9.0)	0.69
Birthweight (kilograms)	3.1 ± 0.7 (1.6 – 4.8)	3.5 ± 0.5 (2.0 – 4.6)	**0.010**
Gestational age (weeks)	38.0 ± 2.0 (33.0 – 41.0)	39.4 ± 1.9 (35.0 – 41.0)	**0.005**
	**Unexposed *n* = 25**	**In Utero exposure *n* = 54**	**Comparison of groups**
	n (%)	n (%)	*P*-value
Sex**: Female**	13 (52%)	34 (63%)	0.46
BMI-for-Age	Mean = 16.5	Mean = 16.7	0.74
Underweight (< 5^th^)	2 (8%)	0 (0%)	
Normal BMI (5-85^th^)	17 (68%)	41 (76%)	
Overweight or obese (≥ 85^th^)	6 (24%)	13 (24%)	
Breastfeeding duration: < **3 months**	6 (24%)	11 (20%)	0.34
Maternal education: > **Year 12**	16 (64%)	40 (74%)	0.56
Maternal smoking during pregnancy	1 (4%)	4 (7%)	0.99
Maternal alcohol consumption during pregnancy	0 (0%)	1 (2%)	0.99
Maternal overall stress: **Mostly stressed**	19 (76%)	35 (65%)	0.06
Maternal fire stress: **Increased a lot**	20 (80%)	43 (81%)	0.34
Maternal asthma	5 (20%)	15 (28%)	0.78
Second hand tobacco smoke	1 (4%)	7 (13%)	0.99
Cold or flu in last 3 weeks	8 (32%)	13 (24%)	0.58
Medication in last 24 h	2 (8%)	8 (15%)	0.49
	median (IQR)	median (IQR)	
Mean ambient background PM_2.5_ (µg/m^3^)	NA	5.1 (4.9 – 5.2)	N/A
Mean average daily PM_2.5_ (µg/m^3^)	N/A	4.2 (2.6 – 14.7)	N/A
Maximum average daily PM_2.5_ (µg/m^3^)	N/A	88 (52 – 225)	N/A

Index of Relative Socio-economic Disadvantage (IRSD) decile, Body mass index (BMI-for age) calculation included gender, age at time of measurement, height and weight, maternal smoking during pregnancy (Y/N), maternal alcohol consumption during pregnancy (Y/N), maternal asthma (Y/N), exposure to second hand smoke (Y/N), cold or flu in the last 3 weeks (Y/N), medication usage in last 24 h (Y/N). Exposure estimates for PM_2.5_ based on methods section *Exposure Estimate.* Unexposed and exposed participant characteristics were compared, continuous outcomes with T-tests and binary outcomes with Fischer’s exact test. *P*-values < 0.05

### Covariate characteristics

Factors including sex, BMI, breastfeeding duration, exposure to second-hand smoke, cold/flu or medication usage in the last 24 h, and maternal factors such as education, smoking during pregnancy, alcohol consumption during pregnancy, overall stress, fire stress, maternal asthma were balanced between the unexposed children and exposed children (Table 1).There was a reasonable sex balance in the sample (59% female), and 24**%** of the children were overweight or obese. The sub-sample of exposed children that had acceptable respiratory measurements had a relatively high socioeconomic status with 74% mothers reporting education of greater than year 12 (Table 1). This group also had one(2%) mother that consumed alcohol during pregnancy, 4(7%) mothers who smoked during pregnancy and 7(13%) were exposed to second-hand smoke. Sixty-five percent of mothers were ‘mostly stressed’ overall during pregnancy, and 81% of mother’s stress ‘increased a lot’ due to the fire. The medians (interquartile ranges) for mean and maximum average PM_2.5_ were 4.2(2.6 – 14.7) and 88(52 – 225) µg/m^3^ respectively for the exposed children (Table 1). These exposure estimates had a background of low ambient levels of PM_2.5_ with a median (interquartile range) of 5.1(4.9 – 5.2) µg/m^3^.

There were some differences in the participant characteristics of the sub-group of children who attended the clinic compared to those who did not. Interestingly, the unexposed children who attended the clinic were lighter at birth, fewer gestational weeks, had fewer mothers that smoked during pregnancy and lower rates of second-hand tobacco smoke exposure (Table S1, Supporting Information). In contrast, exposed children who attended the clinic had more educated mothers, lower rates of second-hand smoke and higher median PM_2.5_ compared to children that did not attend (Table S2, Supporting Information).

### Comparison of respiratory Z-scores by exposure group

**Fig. 2:**
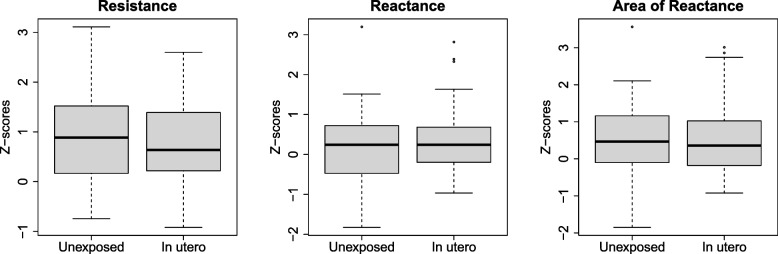
Comparison of mean respiratory Z-scores of unexposed and in utero exposed children

Mean respiratory Z-scores and standard deviation were calculated for each exposure group. Unexposed children had mean (± SD) respiratory R_5_, X_5_, and AX Z-scores of 0.82 ± 0.98, 0.21 ± 1.02, and 0.56 ± 1.09, respectively. Exposed children had mean respiratory R_5,_ X_5_*,* and AX Z-scores 0.74 ± 0.80, 0.29 ± 0.79, and 0.46 ± 0.92, respectively (Fig. 2). There were no statistically significant differences between the respiratory Z-score for the unexposed and exposed children (R_5_ (β = -0.09, 95% CI = -0.50, 0.32), X_5_ (β = 0.09, 95% CI = -0.33, 0.50), or AX (β = -0.10, 95% CI = -0.56, 0.36), Table 2). Similarly, there were no statistically significant differences between respiratory Z-scores in unexposed and exposed children after adjustment for identified covariates (R_5_ (β = -0.06, 95% CI = -0.46, 0.35), X_5_ (β = 0.12, 95% CI = -0.28, 0.52), or AX (β = -0.06, 95% CI = -0.52, 0.39), Table 2).

**Table 2:** Linear regression analysis of respiratory Z-scores and exposure group using pooled imputed models

Association between respiratory z-scores and exposure group				
Z-score	Univariate model		Multivariate model	
	β (95% CI)	P	β (95% CI)	P
R_5_	-0.09 (-0.50, 0.32)	0.68	-0.06 (-0.46, 0.35)	0.78
X_5_	0.09 (-0.33, 0.50)	0.68	0.12 (-0.28, 0.52)	0.55
AX	-0.10 (-0.56, 0.36)	0.68	-0.06 (-0.52, 0.39)	0.79

Outcomes: Resistance at 5 Hz (R_5_), reactance at 5 Hz (X_5_), area under reactance curve (AX)

Multivariate model included the following covariate: maternal education (< or > postsecondary)

### Associations between respiratory function and exposure to PM_2.5_

**Fig. 3:**
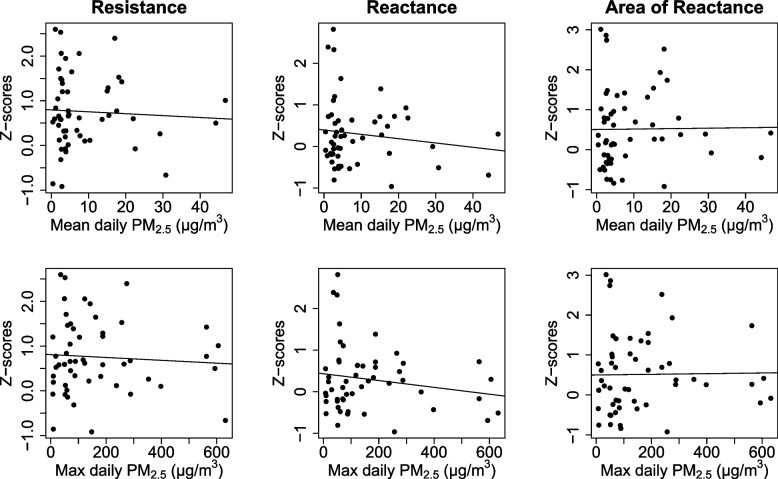
Visualisation of univariate linear regression of association between respiratory function (R_5_, X_5_, AX) and exposure to mean or maximum daily average PM_2.5_. Mine fire PM_2.5_ was assessed in increments of 10 µg/m^3^ and 100 µg/m^3^ for mean maximum average daily exposure, respectively

There were no statistically significant univariate relationships between any of the respiratory Z-score measures and mean average daily PM_2.5_ in the exposed children, R_5_ (β = -0.01, 95% CI = -0.22, 0.20), X_5_ (β = -0.04, 95% CI = -0.24, 0.17), or AX (β = 0.05, 95% CI = -0.19, 0.29), Table 3 and Fig. 3). There was also no association between respiratory Z-scores and maximum average daily PM_2.5_ (R_5_ (β = -0.01, 95% CI = -0.15, 0.12), X_5_ (β = -0.05, 95% CI = -0.18, 0.08), or AX (β = 0.03, 95% CI = -0.12, 0.18), Table 3 and Fig. 3).

**Table 3:** Univariate linear regression analysis, and multivariate analysis adjusting for identified covariates, using pooled imputed models

Association between respiratory z-scores and pm_2.5_ exposure					
	Z-score	Univariate model		Multivariate model	
		β (95% CI)	P	β (95% CI)	P
Mean PM_2.5_ (increments of 10 µg/m^3^)	R_5_	-0.01 (-0.22, -0.20)	0.90	-0.02 (-0.23, 0.19)	0.85
	X_5_	-0.04 (-0.24, 0.17)	0.72	-0.04 (-0.25, 0.16)	0.69
	AX	0.05 (-0.19, 0.29)	0.71	0.04 (-0.20, 0.29)	0.72
Maximum PM_2.5_ (increments of 100 µg/m^3^)	R_5_	-0.01 (-0.15, 0.12)	0.83	-0.01 (-0.14, 0.12)	0.89
	X_5_	-0.05 (-0.18, 0.08)	0.45	-0.05 (-0.18, 0.08)	0.47
	AX	0.03 (-0.12, 0.18)	0.68	0.03 (-0.12, 0.18)	0.67

Outcomes: Resistance at 5 Hz (R_5_), reactance at 5 Hz (X_5_), area of reactance (AX)

Multivariate model included the following covariate: maternal education (< or > postsecondary)

The associations between R_5_, X_5_ and AX and mean average daily PM_2.5_ were not altered by adjustment for covariates (*p* > 0.69 for all comparisons, Table 3) and there was minimal effect on the precision of the estimates. Similarly, the associations between R_5_, X_5_ and AX and maximum average daily PM_2.5_ were not altered by adjustment for covariates (*p* > 0.47 for all comparisons, Table 3).

## Discussion

The health consequences of acute, high-intensity air pollution are relatively unknown, especially in vulnerable populations such as children. This study, which evaluated the long-term effects of in utero exposure to coalmine fire on childhood lung function, found no difference in lung function between unexposed children and children exposed in utero. Similarly, there was no relationship between respiratory Z-scores (R_5_, X_5_ and AX) and PM_2.5_ exposure within the exposed children. Thus, we found no overall association between prenatal exposure to acute, high-intensity air pollution and post-natal lung function.

Research investigating the health implications of prenatal exposure to air pollution has focused on chronic, low-intensity air pollution from urban settings including diesel-exhaust, industrial emissions and emissions from fossil fuel power generation [22]. However, the current study is one of the first to investigate the impact of prenatal exposure to acute, high-intensity air pollution on post-natal lung function against a background of low ambient levels of PM_2.5_. Prenatal exposure to landscape fire smoke has been a growing area of importance, due to the increasing frequency and severity of landscape fires, and has been linked to numerous negative health consequences [22]. Although the chemical characteristics of landscape fires may differ from emissions from a coalmine fire, the intensity and duration of exposure are comparable [22].

Prenatal exposure to landscape fire smoke has been associated with prematurity [23, 24] and lower birth weight [23–25]. However, we observed smaller birthweight and fewer gestational weeks in unexposed children compared to the exposed children. The reasons for this discrepancy are unclear but it is worth noting that the unexposed children that attended the clinic were also smaller and had a lower gestational age than the unexposed children that did not attend the clinic. These factors were not retained in multivariate models based on the outcome of the stepwise regression.

This study was the first to evaluate the long-term effects of prenatal exposure to acute, high-intensity air pollution on childhood lung function. There are a couple of plausible explanations for the null findings. Firstly, PM_2.5_ includes the size fraction of ultrafine particles that can move from the lungs and into the cardiovascular system, cross the placental barrier and enter the foetal circulation [26, 27]. Hence, it is likely that the foetus was directly exposed to PM_2.5_ from the mine fire episode but the duration of exposure may have been too short for any substantial damage to occur [28]. Secondly, there may have been short term changes to lung function in these children, but they disappeared over time due to catch-up growth resulting in no observable changes 7-years after the fire episode [29]. The children exposed in utero were too young to undergo respiratory testing during the previous clinics, in 2017, so this is the first time we had an opportunity to assess this group.

This study has numerous strengths including individual exposure estimates of mine fire emissions and background PM_2.5_ for each participant. Another strength of this study was the use of FOT to assess lung mechanics, providing a more detailed assessment of lung function; particularly in this age group compared to other lung function techniques such as spirometry. The clear exposure windows defined in the ELF cohort are a considerable strength of the study. We can be confident in utero exposed children were exposed to prenatal and not high postnatal PM_2.5_.

However, this study had a number of limitations including the small sample size and potential for bias in the study sample based on the differential characteristics in exposure groups and in those who attended the clinics. The cohort was relatively small at recruitment, as this coalmine fire affected a number of small, rural communities. In order to detect a clinically meaningful change in lung function (0.5 changes in Z score) we required 64 children per group based on a two-group comparison. Therefore, the study may have been underpowered.

There were differences in the sub-group of the exposed children who attended clinical testing and acceptable respiratory measurements compared to exposed children who did not attend (Table S2, Supporting Information). Children from the most severely impacted areas were likely to be more motivated to attend clinical testing. Mothers with higher education attainment were also more likely to understand the value in attending clinics. Higher education attainment and lower rates of second-hand smoking are associated with better lung function outcomes in children [30, 31]. The modest attendance in both exposed and unexposed groups may have reduced the strength of the observed relationships and assessment at this time point may have missed acute, subclinical changes to lung function. The differences in the characteristics of children who attended clinical testing by exposure group may have impacted the representativeness of the sample and affected the translatability of the findings.

Our observations provide encouraging findings, both for the local community and more broadly, as the long-term health consequences of acute high-intensity air pollution are relatively unknown. Further work is needed to understand the long-term implication of prenatal exposure to acute, high-intensity air pollution on lung function at various time points in childhood.

## Availability of data materials

The data that support the findings of this study are available from the corresponding author upon reasonable request.

## Supplementary Information

**Additional file 1:** **Table S1.** Comparison of participant characteristics andcovariates of unexposed participants who had acceptable respiratorymeasurements and those who did not attend clinics or had unacceptable measures.**Table S2.** Comparison of participantcharacteristics and covariates of *inutero* exposed participants who attended clinical testing and had acceptablerespiratory measurements and those who did not attend or did not haveacceptable respiratory measurements. **Table S3.** R packages.

## Abbreviations

AX: Area under the reactance curve
BMI: Body mass index
ELF: Latrobe Early Life Follow-up Study
FEV_1_: Forced expiratory volume in first second
FOT: Forced oscillation technique
FVC: Forced vital capacity
HHS: Hazelwood Health Study
IRSD: Index of relative social disadvantage
PM: Particulate matter
PM_2.5_: Particulate matter with an aerodynamic diameter of less than 2.5 µm
PM_10_: Particulate matter with an aerodynamic diameter of less than 10 µm
R_5_: Resistance at 5 Hz
SD: Standard deviation
X_5_: Reactance at 5 Hz
