# Supplementary material for: No association between in utero exposure to emissions from a coalmine fire and post-natal lung function
Source: BMC Pulm Med. 2023 Apr 14;23:120. doi: 10.1186/s12890-023-02414-7 (PMC10103534; doi:10.1186/s12890-023-02414-7)
Supplement: Supplementary file 1 — Additional file 1: Table S1. Comparison of participant characteristics andcovariates of unexposed participants who had acceptable respiratorymeasurements and those who did not attend clinics or had unacceptable measures.Table S2. Comparison of participantcharacteristics and covariates of inutero exposed participants who attended clinical testing and had acceptablerespiratory measurements and those who did not attend or did not haveacceptable respiratory measurements. Table S3. R packages. [file 12890_2023_2414_MOESM1_ESM.docx]

**SUPPORTING INFORMATION:**

*Respiratory function*

Lung function was evaluated using the forced oscillation technique (FOT) (TremoFlo C-100, Thorasys, Montreal, QC, Canada) according to American Thoracic Society/European Respiratory Society guidelines.^18^ Lung function was assessed in children during quiet tidal breathing while seated with their heads in a neutral position and nose clips in place. Daily field calibrations were conducted with a test load. Standardized Z-scores were calculated for resistance (R_5_) and reactance (X_5_) at a frequency of 5 Hz and the area under the reactance curve (AX).^19^ The Z-scores account for differences in age, height, and gender. Mean Z-scores were calculated for each respiratory measure from three to five acceptable measurements with a coefficient of variation <10%. Measurements were excluded if there was evidence of artefacts due to mouth or tongue movement, glottal closure or talking, swallowing and/or leakage.^31, 32^

*Excluded participants*

There were some differences in the participant characteristics of the sub-group of children who attended the clinic compared to those who did not. Interestingly, the unexposed children who attended the clinic had younger gestational age, smaller birthweight, fewer mothers who smoked during pregnancy and fewer were exposed to second-hand tobacco smoke exposure (Supporting Information, Table S1). The participant characteristics in this sub-group of unexposed children did not differ by IRSD, sex and maternal factors (Supporting Information, Table S1). In contrast, children exposed *in utero* who attended clinic had more educated mothers, lower rates of second-hand smoke and higher median PM_2.5_ (Supporting Information, Table S2). The participant characteristics of the sub-group of children exposed *in utero* had acceptable respiratory measurements did not differ by IRSD, birthweight, gestational age, sex, and maternal factors compared to children that did not have acceptable respiratory measurements or did not attend the clinic (Supporting Information, Table S2).

*Statistical analysis*

Two sets of analyses were conducted to address two separate questions: (1) whether mine fire exposure (a binary indicator; conceived after the mine fire vs *in utero* exposed) was associated the respiratory Z-scores; (2) whether there were any dose-response relationships between fire-related PM_2.5_ exposure (mean daily PM_2.5_ and maximum daily PM_2.5_) and respiratory outcomes among those exposed (children conceived after the mine fire were excluded).

Univariate linear regression models were first used to evaluate the association between exposure to mine fire and respiratory Z-scores (R_5_, X_5_, AX). Possible covariates were identified *a priori* based on our previous work and included; sex, BMI, breastfeeding duration, maternal education, smoking during pregnancy, alcohol consumption during pregnancy, overall stress, fire stress, maternal asthma, exposure to second-hand smoke, cold or flu or medication usage in the last 24 hours.^15^ However, due to the small sample size, these covariates cannot be all controlled in the multivariate analysis. Therefore, step-wise regression models (using R package olsrr version 0.5.3) were used to select covariates for each outcome variable separately for the final multivariate regression model.

Multiple Imputation by Chained Equations (MICE using the mice R package version 3.14.0) with predicted mean matching was used to address missing data in outcomes and all possible covariates. 20 imputed datasets were generated. The imputed datasets were used for estimating regression coefficients with linear regression models carried out in each imputed dataset and pooled with Rubin’s rule.^20^ The imputed datasets were also used in stepwise variable selection. Covariates were selected if they were retained in 80% of stepwise models across imputed datasets and pooled with Rubin’s rule to calculate estimated regression coefficients in the linear regression models (Table S3, Supporting Information).^20^

Normality of the residuals from the models were assessed by visual inspection and Shapiro-Wilk tests to ensure the validity of the assumption of linearity. R Studio Version 4.1.3 was used for the statistical analysis and all packages (Supporting Information, Table S3). Summary data are reported as means (SD) and ranges. Beta (β)-coefficients, 95% confidence intervals and p values are reported for all regression analyses.

**SUPPORTING TABLES:**

**Table S1: Comparison of participant characteristics and covariates of unexposed participants who had acceptable respiratory measurements and those who did not attend clinics or had unacceptable measures.**

|  | **Acceptable respiratory measurements n=25** | | **Unacceptable or did not attend clinics n=147** | | **Comparison of groups** |
| --- | --- | --- | --- | --- | --- |
|  | mean ± sd (range) | | mean ± sd (range) | | P-value |
| IRSD decile | 3.5 ± 3.2 (1.0 – 9.0) | | 3.7 ± 3.0 (1.0 – 10.0) | | 0.78 |
| Birthweight (kilograms) | 3.1 ± 0.7 (1.6 – 4.8) | | 3.4 ± 0.7 (1.5 – 5.6) | | **0.041** |
| Gestational age (weeks) | 38.0 ± 2.0 (33.0 – 41.0) | | 39.3 ± 2.0 (32.0 – 42.0) | | **0.005** |
|  | | **Acceptable respiratory measurements n=25** | | **Unacceptable or did not attend n=147** | **Comparison of groups** |
|  | | n (%) | | n (%) | P-value |
| Sex**: Female** | | 13 (52%) | | 66 (45%) | 0.51 |
| Breastfeeding duration: **<3months** | | 6 (24%) | | 47 (32%) | 0.42 |
| Maternal education: >**Year 12** | | 16 (64%) | | 80 (54%) | 0.27 |
| Maternal smoking during pregnancy | | 1 (4%) | | 35 (24%) | **0.029** |
| Maternal alcohol consumption during pregnancy | | 0 (0%) | | 2 (1%) | 0.99 |
| Maternal overall stress: **Mostly stressed** | | 19 (76%) | | 90 (61%) | 0.11 |
| Maternal fire stress: **Increased a lot** | | 20 (80%) | | 85 (58%) | 0.08 |
| Maternal asthma | | 5 (20%) | | 36 (25%) | 0.99 |
| Second hand tobacco smoke | | 1 (4%) | | 48 (33%) | **0.005** |

Legend: Index of Relative Socio-economic Disadvantage (IRSD) decile, Body mass index (BMI-for age) calculation included gender, age at time of measurement, height and weight, maternal smoking during pregnancy (Y/N), maternal alcohol consumption during pregnancy (Y/N), maternal asthma (Y/N), exposure to second hand smoke (Y/N), cold or flu in the last 3 weeks (Y/N), medication usage in last 24 hours (Y/N). Unexposed and exposed participant characteristics were compared, continuous outcomes with T-tests and binary outcomes with Fischer’s exact test. **P-values <0.05**

**Table S2: Comparison of participant characteristics and covariates of *in utero* exposed participants who attended clinical testing and had acceptable respiratory measurements and those who did not attend or did not have acceptable respiratory measurements.**

|  | **Acceptable respiratory measurements n=54** | | **Unacceptable or did not attend clinics n=128** | | **Comparison of groups** |
| --- | --- | --- | --- | --- | --- |
|  | mean ± sd (range) | | mean ± sd (range) | | P-value |
| IRSD decile | 3.3 ± 2.6 (1.0 – 9.0) | | 3.9 ± 3.0 (1.0 – 10.0) | | 0.26 |
| Birthweight (kilograms) | 3.5 ± 0.5 (2.0 – 4.6) | | 3.5 ± 0.7 (1.2 – 5.3) | | 0.84 |
| Gestational age (weeks) | 39.4 ± 1.9 (35.0 – 41.0) | | 39.1 ± 2.0 (30.0 – 42.0) | | 0.25 |
|  | | **Acceptable respiratory measurements n=54** | | **Unacceptable or did not attend clinics n=128** | **Comparison of groups** |
|  | | n (%) | | n (%) | P-value |
| Sex**: Female** | | 34 (63%) | | 60 (47%) | 0.05 |
| Breastfeeding: **<3months** | | 11 (20%) | | 34 (27%) | 0.25 |
| Maternal education: >**Year 12** | | 40 (74%) | | 69 (54%) | **0.006** |
| Maternal smoking during pregnancy | | 4 (7%) | | 19 (15%) | 0.16 |
| Maternal alcohol consumption during pregnancy | | 1 (2%) | | 2 (2%) | 0.99 |
| Maternal overall stress: **Mostly stressed** | | 35 (65%) | | 77 (60%) | 0.86 |
| Maternal fire stress: **Increased a lot** | | 43 (80%) | | 91 (71%) | 0.60 |
| Maternal asthma | | 15 (28%) | | 47 (37%) | 0.23 |
| Second hand smoke | | 7 (13%) | | 41 (32%) | **0.009** |
|  | | median (IQR) | | median (IQR) |  |
| Mean ambient background PM_2.5_ | | 5.1 (4.9 – 5.2) | | 5.1 (4.9 – 5.3) | 0.16 |
| Mean average daily PM_2.5_ (µg/m^3^) | | 4.2 (2.6 – 14.7) | | 2.8 (1.9 – 7.8) | 0.25 |
| Maximum average daily PM_2.5_ (µg/m^3^) | | 88 (52 – 225) | | 75 (52 – 147) | 0.36 |

Legend: Index of Relative Socio-economic Disadvantage (IRSD) decile, Body mass index (BMI-for age) calculation included gender, age at time of measurement, height and weight, maternal smoking during pregnancy (Y/N), maternal alcohol consumption during pregnancy (Y/N), maternal asthma (Y/N), exposure to second hand smoke (Y/N), cold or flu in the last 3 weeks (Y/N), medication usage in last 24 hours (Y/N). Exposure estimates for PM_2.5_ based on methods section *Exposure Estimate.* Unexposed and exposed participant characteristics were compared, continuous outcomes with T-tests and binary outcomes with Fischer’s exact test. **P-values <0.05**

**Table S3: R packages**

| **Package name** | **Version** | **Maintainer** |
| --- | --- | --- |
| dplyr | 1.0.8 | Hadley Wickham <hadley@rstudio.com> |
| janitor | 2.1.0 | Sam Firke <samuel.firke@gmail.com> |
| MASS | 7.3.55 | Brian Ripley <ripley@stats.ox.ac.uk> |
| mice | 3.14.0 | Stef van Buuren <stef.vanbuuren@tno.nl> |
| olsrr | 0.5.3 | Aravind Hebbali <hebbali.aravind@gmail.com> |
| pacman | 0.5.1 | Tyler Rinker <tyler.rinker@gmail.com> |
| PerformanceAnalytics | 2.0.4 | Brian G. Peterson <brian@braverock.com> |
| ranger | 0.13.1 | Marvin N. Wright <cran@wrig.de> |
| readxl | 1.4.0 | Jennifer Bryan <jenny@rstudio.com> |
| xts | 0.12.1 | Joshua M. Ulrich <josh.m.ulrich@gmail.com> |
| zoo | 1.8.9 | Achim Zeileis <Achim.Zeileis@R-project.org> |
